# Supplementary material for: Decoding exon inclusion in the human brain reveals more divergent splicing mechanisms in neurons than glia
Source: Genome Biol. 2026 Feb 28;27:119. doi: 10.1186/s13059-026-04015-z (PMC13059442; doi:10.1186/s13059-026-04015-z)
Supplement: Supplementary file 1 — Additional file 1. TF-MoDISco results after filtering for strand-specificity. [file 13059_2026_4015_MOESM1_ESM.pdf]

pattern	num_seqlets	modisco_cwm_fwd	modisco_cwm_rev	match0	qval0	match0_logo	match1	qval1	match1_logo	match2	qval2	match2_logo
pos_patterns.pattern_0	2806			TRA2A_7mer_logo0	1.000000		PABPN1_7mer_logoM148	1.000000		tra2_7mer_logoM076	1.000000	
pos_patterns.pattern_1	1415			PCBP1_7mer_logoM177	1.000000		DAZAP1_7mer_logoM013	1.000000		Hrb27C_7mer_logoM028	1.000000	
pos_patterns.pattern_2	1120			TVAG514790_7mer_logoM204	0.012887		PABPC4_7mer_logoM042	0.012887		gw184451_7mer_logoM198	0.016268	
pos_patterns.pattern_3	983			HNRNPC_7mer_logo0	0.025064		Sxl_7mer_logoM114	0.031924		gw184451_7mer_logoM198	0.046902	
pos_patterns.pattern_4	977			RBM45_7mer_logo5	0.413316		Rsf1_7mer_logoM059	0.413316		CG2950_7mer_logoM007	1.000000	
pos_patterns.pattern_5	901			TAF15_7mer_logo0	0.012774		HNRNPA2B1_7mer_logo0	0.019606		SRSF8_7mer_logo1	0.019606	
pos_patterns.pattern_6	640			KHDRBS2_7mer_logo1	0.454750		KHDRBS3_7mer_logo0	0.454750		RBMS3_7mer_logo2	0.454750	
pos_patterns.pattern_7	614			IGF2BP1_7mer_logo1	0.793093		QKI_7mer_logoM046	0.793093		how_7mer_logoM113	0.793093	
pos_patterns.pattern_8	547			TRA2A_7mer_logo1	0.063024		PUF60_7mer_logo0	0.063024		PABPC4_7mer_logoM042	0.063024	
pos_patterns.pattern_9	394			B52_7mer_logoM126	0.022741		HNRNPF_7mer_logo1	0.022741		HNRNPH2_7mer_logoM151	0.022741	
pos_patterns.pattern_10	360			YB1_7mer_logoM111	0.852294		IGF2BP2_7mer_logo3	1.000000		YB1_7mer_logoM081	1.000000	
pos_patterns.pattern_11	326			Hrb87F_7mer_logoM029	0.005667		Hrb98DE_7mer_logoM092	0.005667		Hrb98DE_7mer_logoM093	0.005667	
pos_patterns.pattern_12	276			KHDRBS3_7mer_logo0	0.245746		RBMS3_7mer_logo2	0.245746		shep_7mer_logoM166	0.290158	
pos_patterns.pattern_13	221			RBM4B_7mer_logo2	1.000000		PCBP1_7mer_logo3	1.000000		RBM22_7mer_logo0	1.000000	
pos_patterns.pattern_14	206			SFPQ_7mer_logo0	0.737892		RBM45_7mer_logo2	0.737892		pum_7mer_logoM101	1.000000	
pos_patterns.pattern_15	163			Hrb98DE_7mer_logoM091	0.410690		HNRNPF_7mer_logo1	0.410690		DAZAP1_7mer_logoM013	0.410690	
pos_patterns.pattern_16	143			QKI_7mer_logoM046	0.063280		how_7mer_logoM113	0.063280		LmjF180180_7mer_logoM187	0.208011	
pos_patterns.pattern_17	137			RBM25_7mer_logo2	0.034396		PUM1_7mer_logo1	0.231354		EWSR1_7mer_logo0	0.247968	
pos_patterns.pattern_18	132			TAF15_7mer_logo1	0.590264		HNRNPH2_7mer_logoM151	0.590264		HNRNPF_7mer_logo0	0.909467	
pos_patterns.pattern_19	128			PCBP1_7mer_logo3	1.000000		PCBP4_7mer_logo2	1.000000		RBM22_7mer_logo0	1.000000	
pos_patterns.pattern_20	111			TAF15_7mer_logo0	0.107660		SRSF8_7mer_logo1	0.107660		RBM25_7mer_logo2	0.163125	
pos_patterns.pattern_21	109			NCU02404_7mer_logoM206	0.236015		tra2_7mer_logoM076	0.236015		SRSF1_7mer_logoM103	0.236015	

pattern	num_seqlets	modisco_cwm_fwd	modisco_cwm_rev	match0	qval0	match0_logo	match1	qval1	match1_logo	match2	qval2	match2_logo
pos_patterns.pattern_22	87			TRA2A_7mer_logo1	1.000000		TRA2A_7mer_logo0	1.000000		YBX2_7mer_logoM082	1.000000	
pos_patterns.pattern_23	61			SF1_7mer_logo2	1.000000		KHDRBS3_7mer_logo3	1.000000		ELAVL4_7mer_logo1	1.000000	
pos_patterns.pattern_24	59			RBM25_7mer_logo0	0.104614		HNRNPA2B1_7mer_logo0	0.104614		HNRNPH2_7mer_logoM151	0.104614	
pos_patterns.pattern_25	55			DAZAP1_7mer_logo2	0.940224		SNRPA_7mer_logoM348	0.940224		HNRNPA0_7mer_logo3	0.940224	
pos_patterns.pattern_26	52			PCBP1_7mer_logo1	0.425954		PCBP2_7mer_logo2	0.536159		PCBP4_7mer_logo1	0.536159	
pos_patterns.pattern_27	51			SAMD4A_7mer_logoM061	0.999996		RC3H1_7mer_logo1	0.999996		pUf68_7mer_logoM132	0.999996	
pos_patterns.pattern_28	43			TRNAU1AP_7mer_logo1	1.000000		BOLL_7mer_logo3	1.000000		MSI1_7mer_logo1	1.000000	
pos_patterns.pattern_29	29			tra2_7mer_logoM076	1.000000		TRA2A_7mer_logo0	1.000000		NaN	NaN	
pos_patterns.pattern_30	27			EWSR1_7mer_logo3	1.000000		TRA2A_7mer_logo1	1.000000		SFPQ_7mer_logo0	1.000000	
neg_patterns.pattern_0	1873			NaN	NaN		NaN	NaN		NaN	NaN	
neg_patterns.pattern_1	915			HNRNPH2_7mer_logoM151	0.010881		HNRNPF_7mer_logo0	0.016703		RBM23_7mer_logo0	0.016703	
neg_patterns.pattern_2	883			TAF15_7mer_logo0	0.006430		PCBP1_7mer_logo0	0.016483		SRSF8_7mer_logo1	0.016483	
neg_patterns.pattern_3	743			NaN	NaN		NaN	NaN		NaN	NaN	
neg_patterns.pattern_4	699			Tbg97295210_7mer_logoM216	0.008832		Fusip1_7mer_logoM087	0.017973		PUF60_7mer_logo0	0.017973	
neg_patterns.pattern_5	628			Sxl_7mer_logoM114	0.018752		TVAG514790_7mer_logoM204	0.018752		PABPC4_7mer_logoM042	0.018752	
neg_patterns.pattern_6	540			PTBP3_7mer_logo0	1.000000		KHDRBS3_7mer_logo3	1.000000		PTBP3_7mer_logo1	1.000000	
neg_patterns.pattern_7	540			HNRNPH2_7mer_logoM151	0.014835		HNRNPF_7mer_logo0	0.014835		HNRNPF_7mer_logo1	0.014835	
neg_patterns.pattern_8	400			TRNAU1AP_7mer_logo2	0.592513		KHDRBS3_7mer_logo0	0.592513		RBM15B_7mer_logo1	0.592513	
neg_patterns.pattern_9	386			PUF60_7mer_logo0	0.060297		HNRNPC_7mer_logo0	0.060297		PABPC4_7mer_logoM042	0.060297	
neg_patterns.pattern_10	358			TAF15_7mer_logo0	0.217791		SRSF8_7mer_logo1	0.217791		SFPQ_7mer_logo3	0.217791	
neg_patterns.pattern_11	311			HNRNPC_7mer_logo0	0.040238		Sxl_7mer_logoM114	0.040438		ELAVL1_7mer_logoM232	0.040438	
neg_patterns.pattern_12	295			CPEB1_7mer_logo0	0.671904		DAZ3_7mer_logo3	0.671904		KHDRBS2_7mer_logo1	0.671904	

pattern	num_seqlets	modisco_cwm_fwd	modisco_cwm_rev	match0	qval0	match0_logo	match1	qval1	match1_logo	match2	qval2	match2_logo
neg_patterns.pattern_13	292			CG2931_7mer_logoM138	0.109767		BOLL_7mer_logo2	0.125769		RBM42_7mer_logoM142	0.125769	
neg_patterns.pattern_14	269			HNRNPC_7mer_logo0	0.016414		Sxl_7mer_logoM114	0.016414		TVAG514790_7mer_logoM204	0.016414	
neg_patterns.pattern_15	243			HNRNPF_7mer_logo1	1.000000		EWSR1_7mer_logo3	1.000000		RBM25_7mer_logo2	1.000000	
neg_patterns.pattern_16	227			TIA1_7mer_logo0	0.034657		KHDRBS3_7mer_logo1	0.036074		ELAVL4_7mer_logo0	0.047855	
neg_patterns.pattern_17	220			exc7_7mer_logoM014	0.659310		ELAVL4_7mer_logo2	0.820726		TRA2A_7mer_logo0	0.820726	
neg_patterns.pattern_18	204			HNRNPA2B1_7mer_logo0	0.018745		EWSR1_7mer_logo3	0.018745		HNRNPH2_7mer_logoM151	0.018745	
neg_patterns.pattern_19	193			shep_7mer_logoM066	0.454675		KHDRBS2_7mer_logo0	0.454675		shep_7mer_logoM166	0.454675	
neg_patterns.pattern_20	192			A1CF_7mer_logo0	0.237324		TRNAU1AP_7mer_logo1	0.237324		HNRNPD_7mer_logo0	0.237324	
neg_patterns.pattern_21	178			RBM25_7mer_logo1	0.985434		PCBP2_7mer_logo1	0.985434		RBM23_7mer_logo0	0.985434	
neg_patterns.pattern_22	146			KHDRBS3_7mer_logo0	1.000000		RBMS3_7mer_logo2	1.000000		CELF1_7mer_logo2	1.000000	
neg_patterns.pattern_23	140			ELAVL1_7mer_logoM232	0.001467		gw184451_7mer_logoM198	0.001467		HNRNPC_7mer_logo0	0.008244	
neg_patterns.pattern_24	139			tra2_7mer_logoM076	0.080765		TRA2A_7mer_logo0	0.097847		shep_7mer_logoM165	0.760849	
neg_patterns.pattern_25	128			PPRC1_7mer_logoM044	0.000002		RBM4_7mer_logoM050	0.005436		RBM4B_7mer_logo0	0.005436	
neg_patterns.pattern_26	120			MSI1_7mer_logoM040	1.000000		MSI1_7mer_logoM167	1.000000		RBM25_7mer_logo2	1.000000	
neg_patterns.pattern_27	100			SRSF5_7mer_logo2	0.637969		TAF15_7mer_logo1	0.637969		PCBP2_7mer_logo0	0.637969	
neg_patterns.pattern_28	94			SFPQ_7mer_logo4	0.205755		CNOT4_7mer_logo2	0.205755		PCBP1_7mer_logo0	0.205755	
neg_patterns.pattern_29	71			EIF4G2_7mer_logo3	0.031424		PPRC1_7mer_logoM044	0.035493		SFPQ_7mer_logo4	0.035493	
neg_patterns.pattern_30	62			RBM41_7mer_logoM051	1.000000		RC3H1_7mer_logo0	1.000000		shep_7mer_logoM165	1.000000	
neg_patterns.pattern_31	61			FMR1_7mer_logoM016	0.366338		FXR2_7mer_logoM020	0.366338		SRSF2_7mer_logo3	0.952385	
neg_patterns.pattern_32	60			CG2931_7mer_logoM138	0.158612		RBM42_7mer_logoM142	0.315635		BOLL_7mer_logo2	1.000000	
neg_patterns.pattern_33	60			PPRC1_7mer_logoM044	0.033704		SRSF5_7mer_logo2	0.033704		TAF15_7mer_logo2	0.033704	
neg_patterns.pattern_34	57			Hrb27C_7mer_logoM090	0.287879		Hrb27C_7mer_logoM028	0.287879		ANIA04546_7mer_logoM226	0.287879	

pattern	num_seqlets	modisco_cwm_fwd	modisco_cwm_rev	match0	qval0	match0_logo	match1	qval1	match1_logo	match2	qval2	match2_logo
neg_patterns.pattern_35	52		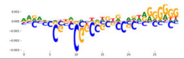	PCBP1_7mer_logo0	0.215737	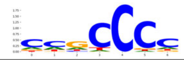	PPRC1_7mer_logoM044	0.215737	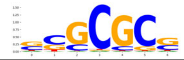	TAF15_7mer_logo1	0.215737	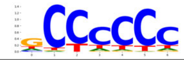
neg_patterns.pattern_36	48	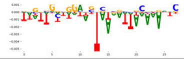		TRNAU1AP_7mer_logo1	0.348772	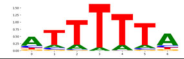	NUPL2_7mer_logo0	0.657776	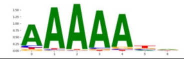	TRNAU1AP_7mer_logo0	0.657776	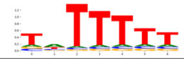
neg_patterns.pattern_37	45	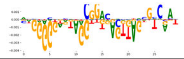		RBM6_7mer_logo3	1.000000	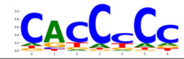	SRSF5_7mer_logo4	1.000000	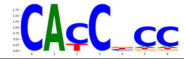	NaN	NaN	
neg_patterns.pattern_38	36	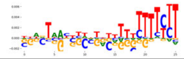		SRSF5_7mer_logo4	0.349634	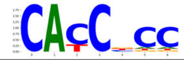	tra2_7mer_logoM076	0.349634	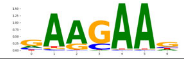	PTBP1_7mer_logoM227	0.349634	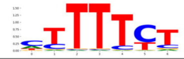
neg_patterns.pattern_39	35	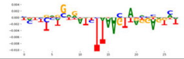		exc7_7mer_logoM014	0.996656	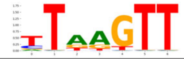	DAZAP1_7mer_logo2	0.996656	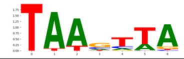	ELAVL4_7mer_logo2	1.000000	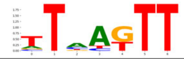
neg_patterns.pattern_41	30	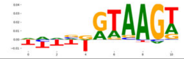		SFPQ_7mer_logo0	0.321676	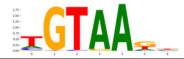	QKI_7mer_logoM046	0.613013	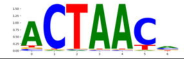	how_7mer_logoM113	0.613013	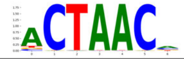
neg_patterns.pattern_43	26	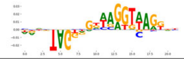		NCU02404_7mer_logoM206	0.565431	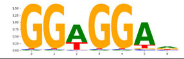	HNRNPF_7mer_logo2	0.565431	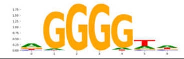	LIN28A_7mer_logoM035	0.565431	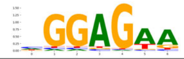
