# Supplementary material for: Decoding exon inclusion in the human brain reveals more divergent splicing mechanisms in neurons than glia
Source: Genome Biol. 2026 Feb 28;27:119. doi: 10.1186/s13059-026-04015-z (PMC13059442; doi:10.1186/s13059-026-04015-z)
Supplement: Supplementary file 2 — Additional file 2. [file 13059_2026_4015_MOESM2_ESM.pdf]

Supplementary data for

Decoding exon inclusion in the human brain reveals more divergent splicing mechanisms in neurons than glia

Lieke Michielsen[1,2,3,4], Justine Hsu[3,4], Anoushka Joglekar[3,4,5], Natan Belchikov[3,4], Marcel J.T. Reinders[1,2], Hagen U. Tilgner[3,4,+], Ahmed Mahfouz[1,2,+]

[1]Department of Human Genetics, Leiden University Medical Center, Leiden, NL
[2]Delft Bioinformatics Lab, Delft University of Technology, Delft, NL
[3]Center for Neurogenetics, Weill Cornell Medicine, New York, NY, USA
[4]Feil Family Brain and Mind Research Institute, Weill Cornell Medicine, New York, NY, USA
[5]New York Genome Center, New York, NY, USA

+ Corresponding authors: Ahmed Mahfouz (a.mahfouz@lumc.nl) and Hagen Tilgner (hut2006@med.cornell.edu)

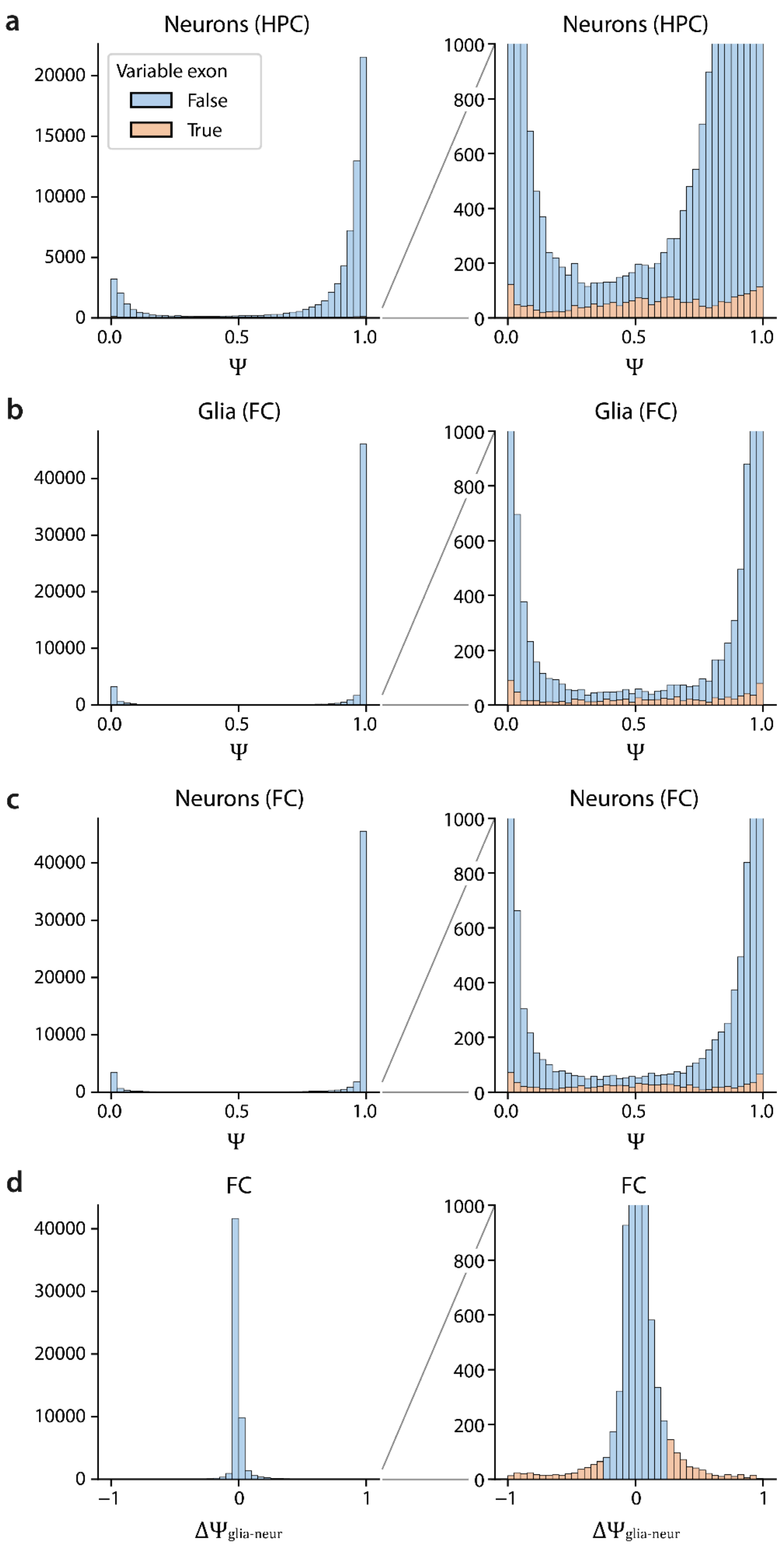

Figure S1 Distribution of **a)** Ψ of neurons in the hippocampus, **b)** Ψ of glia in the frontal cortex, **c)** Ψ of neurons in the frontal cortex, **d)** $\Delta\Psi_{glia-neur}$ in the frontal cortex

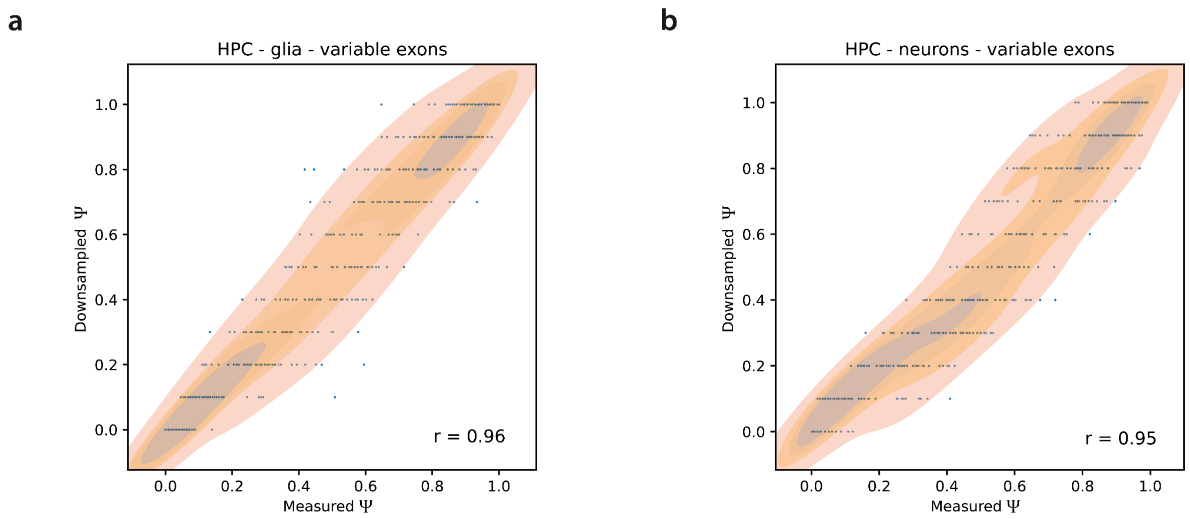

Figure S2 Scatterplot comparing the Ψ values calculated using all reads and the downsampled reads. r = Pearson correlation between measured and downsampled Ψ values.

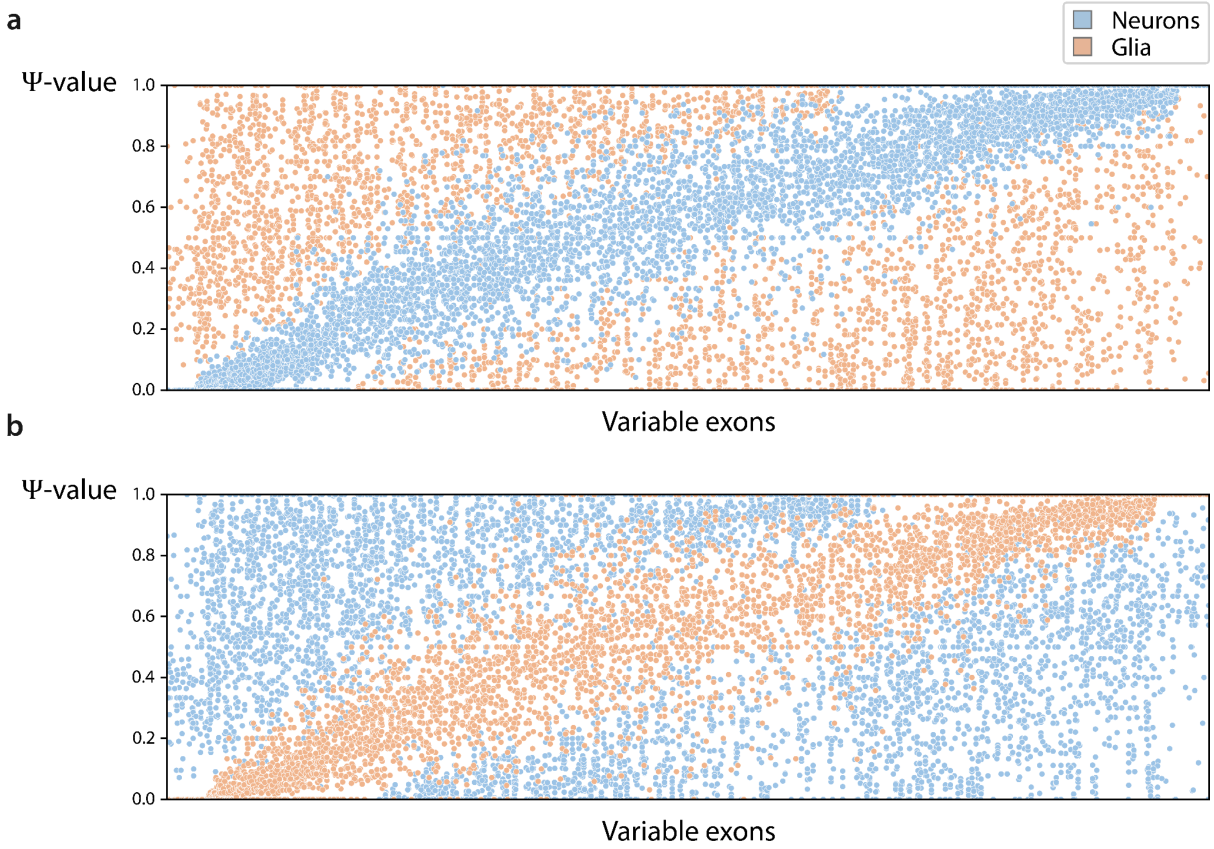

Figure S3 Ψ values per individual for the variable exons in neurons and glia. Every column in the plot represents one variable exon. The variable exons are sorted based on the average Ψ value in **a)** neurons and **b)** glia.

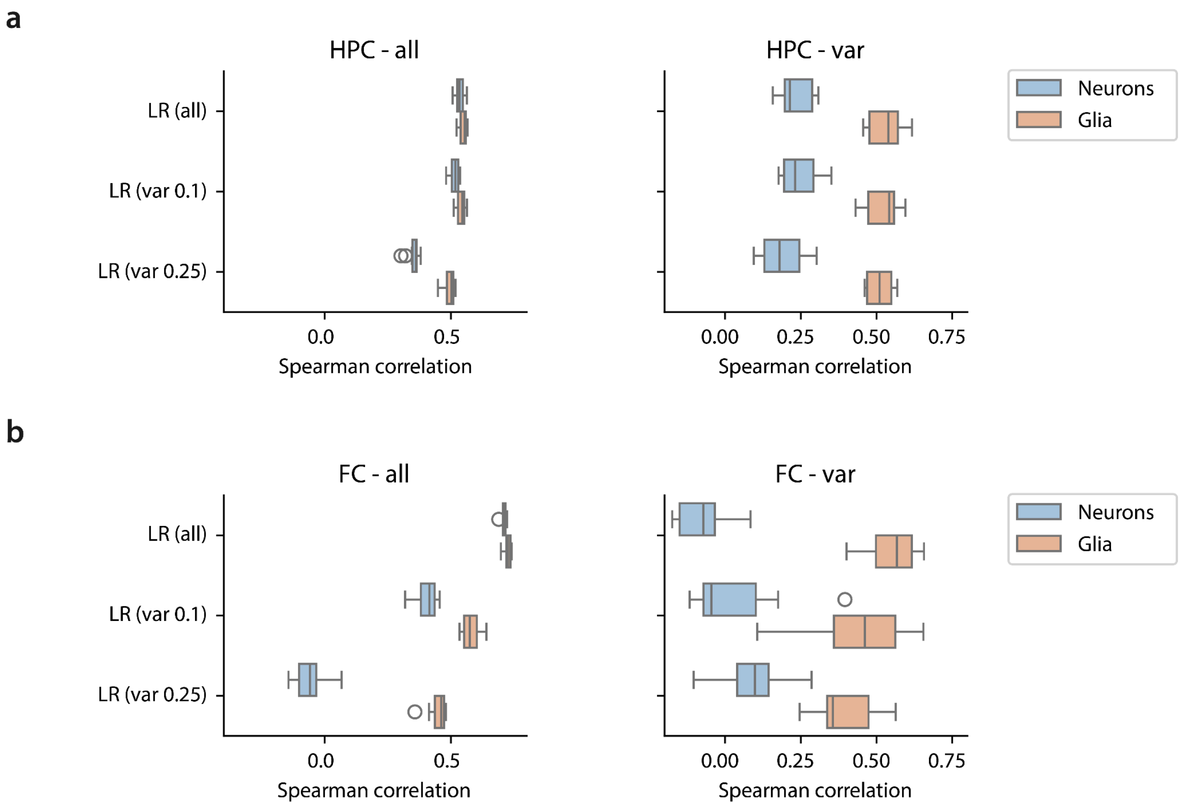

Figure S4 Performance of the different logistic regression models during 10-fold cross-validation on all exons and the variable exons in glia and neurons in the frontal cortex.

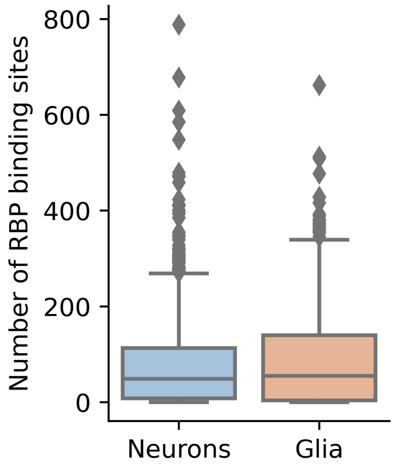

Figure S5 Number of RBP binding sites in variable exons with a higher Ψ value in neurons and glia respectively.

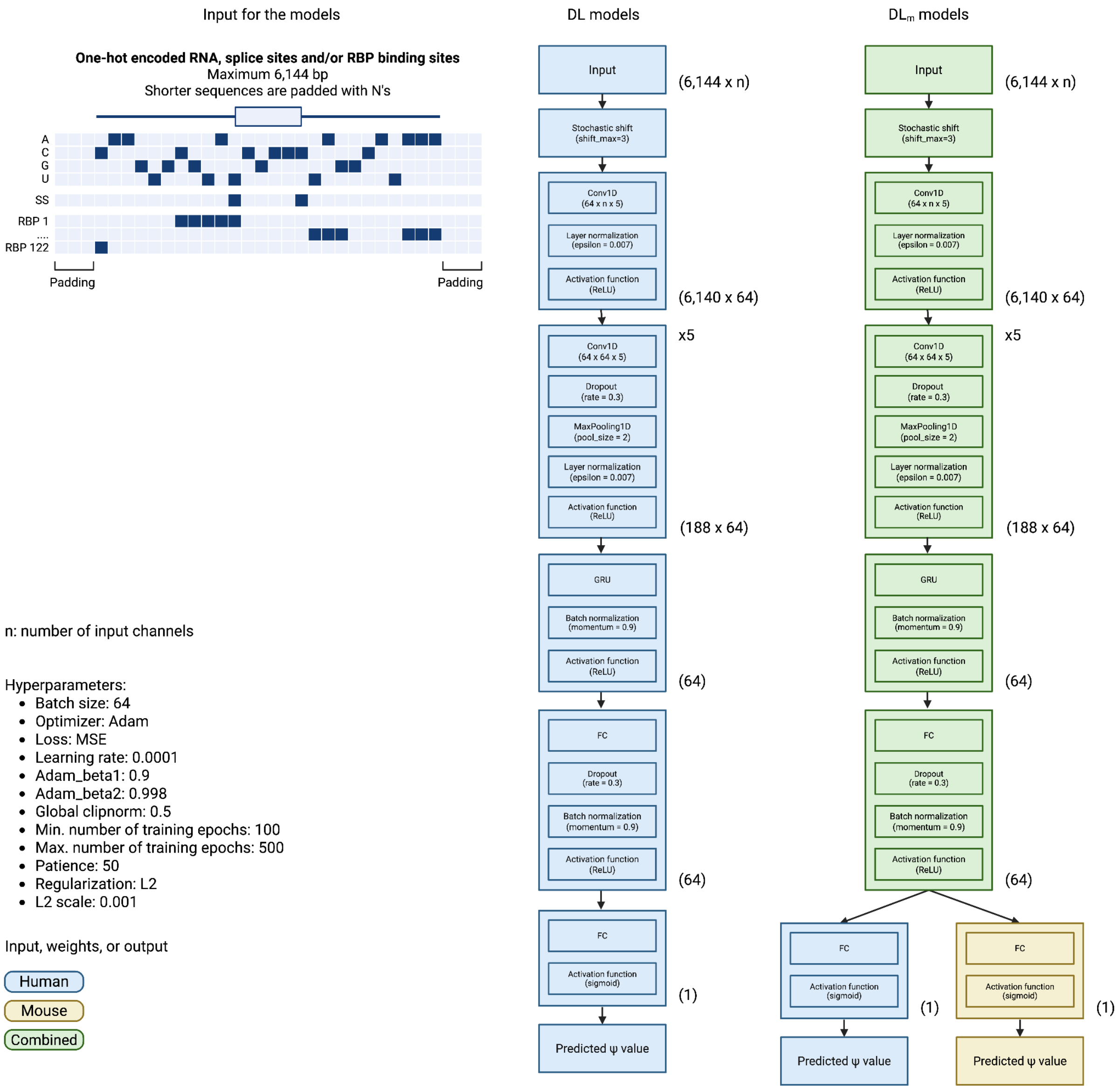

Figure S6 Architecture of the DL and DL_m models. The tuples on the right side of the blocks indicate the output shape.

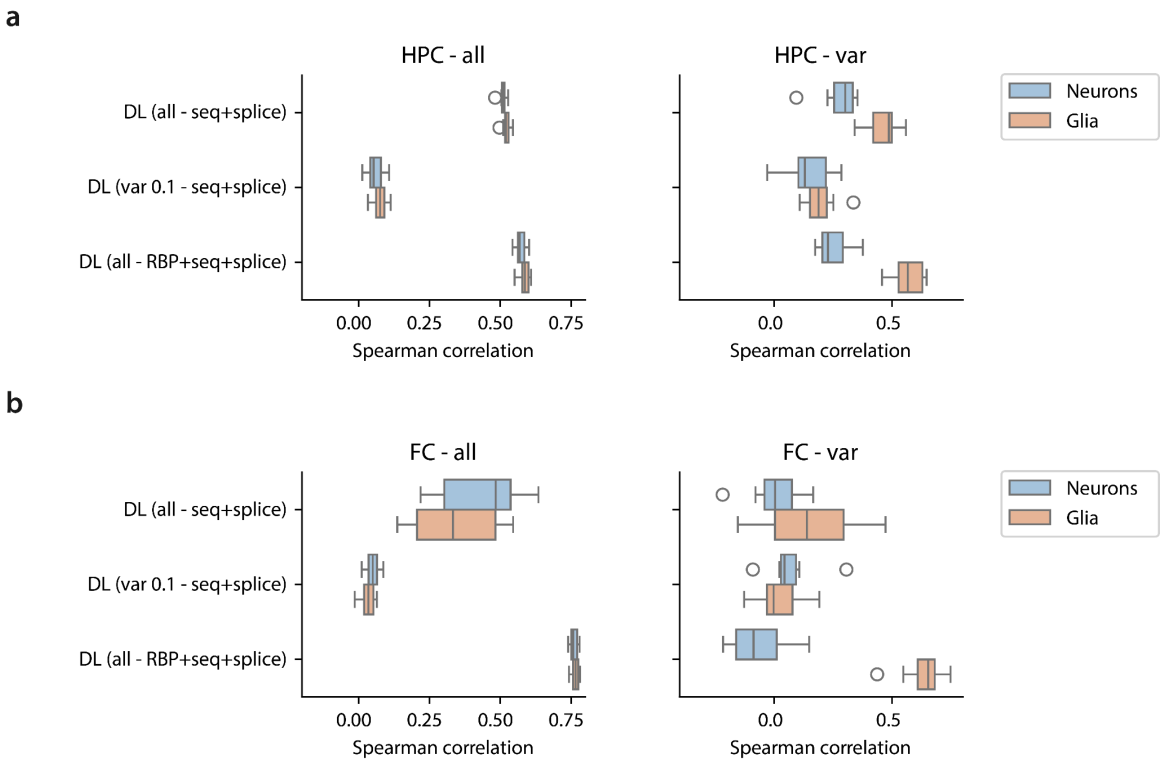

Figure S7 Performance of the different deep learning models during 10-fold cross-validation on all exons and the variable exons in glia and neurons in the frontal cortex.

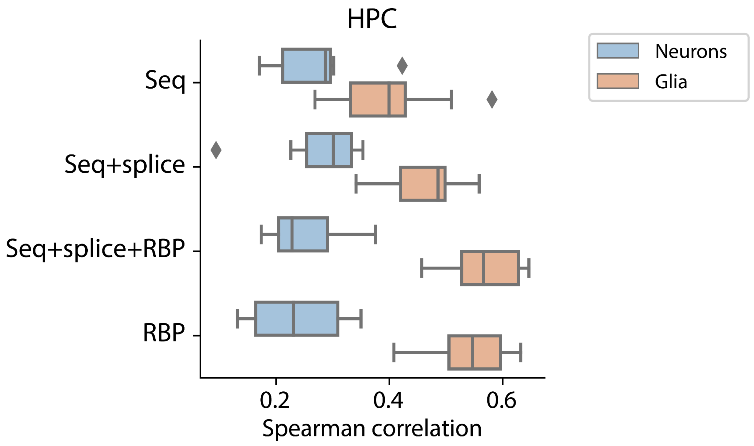

Figure S8 Performance of the DL_{all} models during the 10-fold cross-validation on neurons and glia in the HPC when trained using different input features.

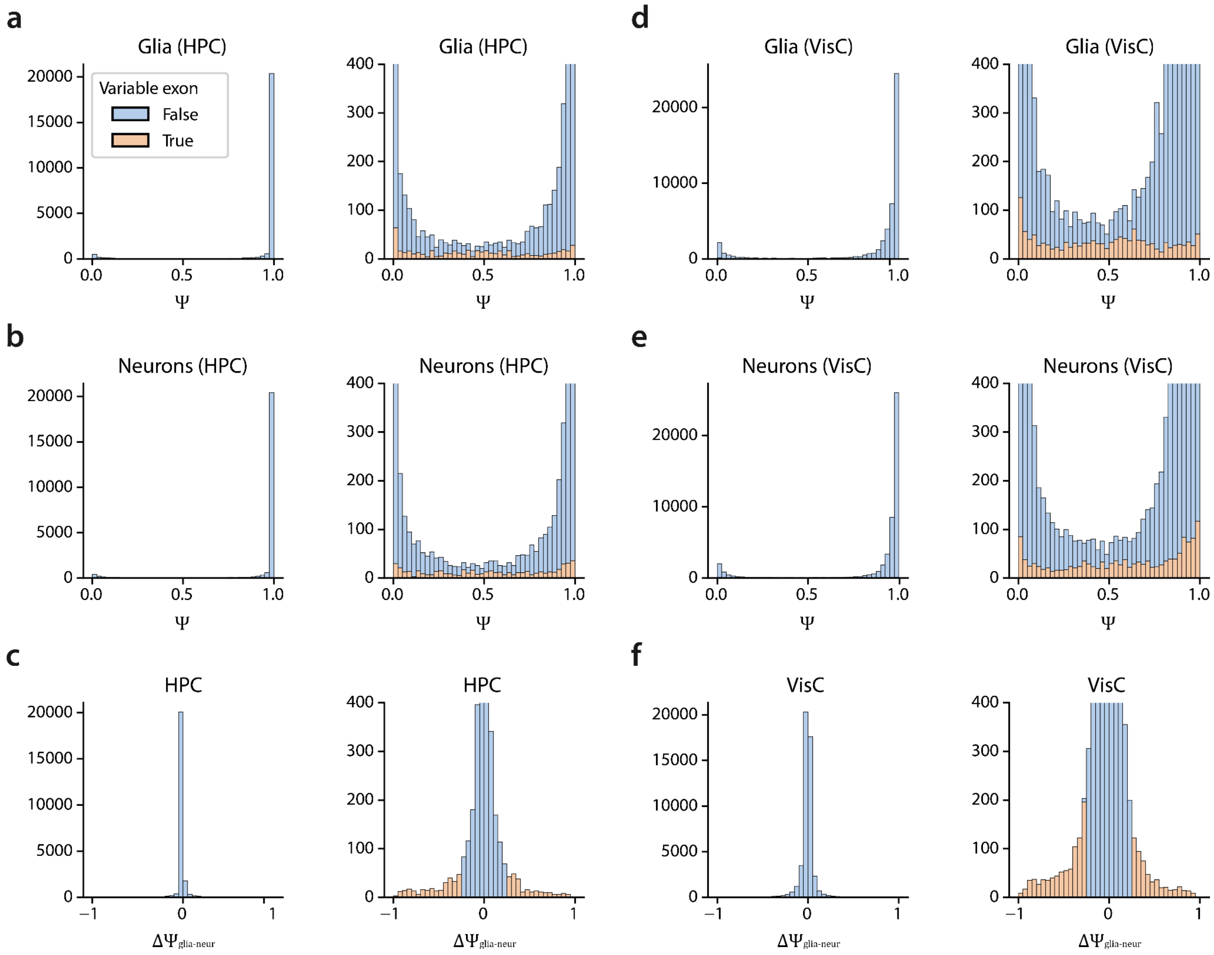

Figure S9 Distribution of Ψ of **a,d)** glia and **b,e)** neurons in the **a-b)** hippocampus and **d-e)** visual cortex in mouse, **c,f)** Distribution of ΔΨ in the hippocampus and visual cortex in mouse. In every panel, the right panel is a zoomed in version of the left plot to better show the distribution of the variable exons.

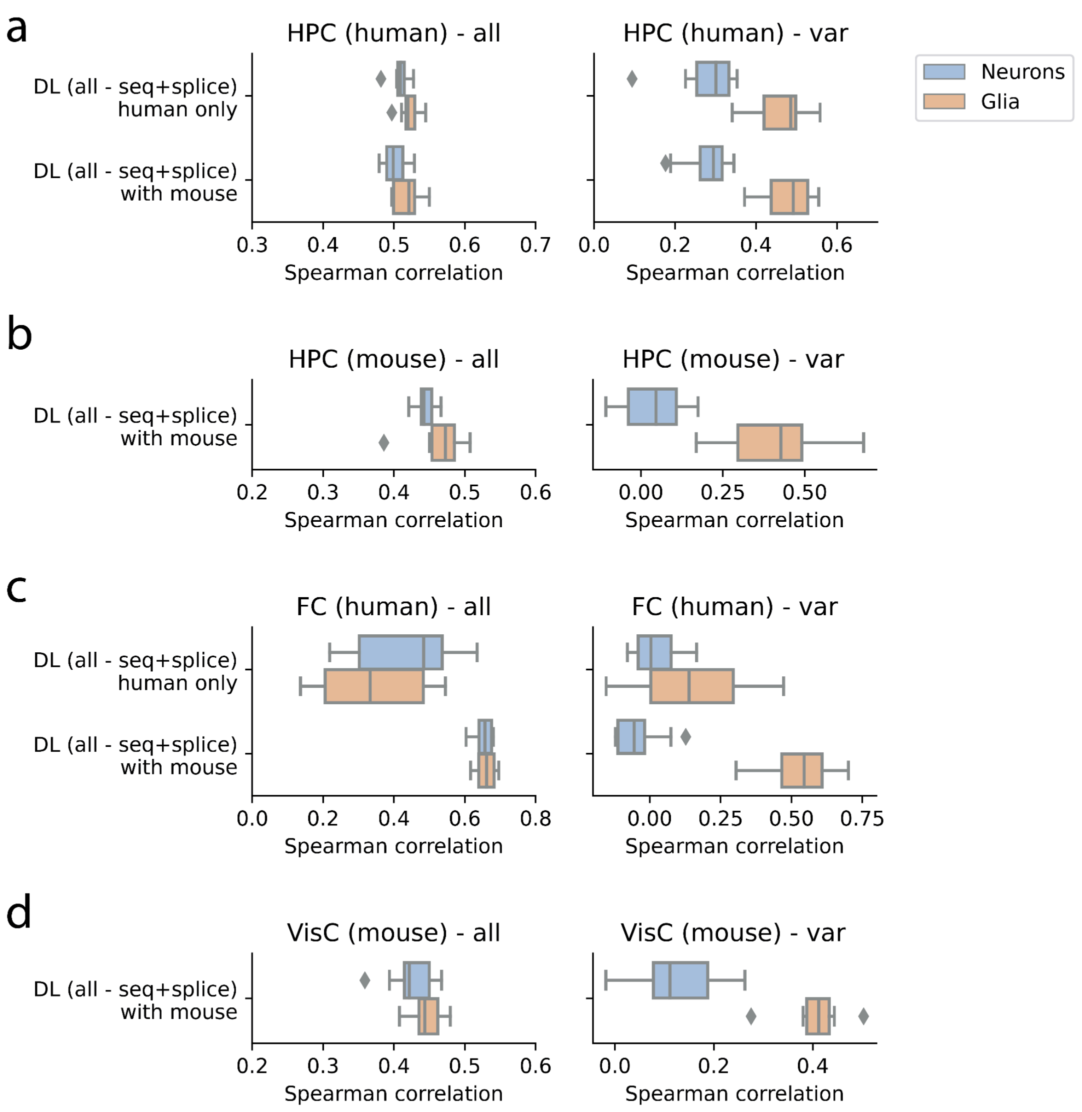

Figure S10 Performance of $DL_{all\text{-}seq}$ trained on human and mouse data during the 10-fold cross-validation on variable **a,c)** human and **b,d)** mouse exons from neurons and glia in the HPC and FC/visual cortex.

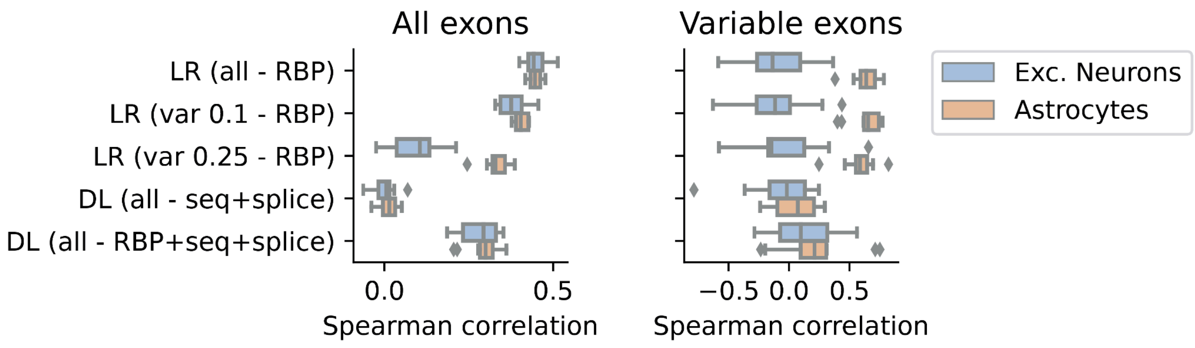

Figure S11 Performance of LR and DL models on all exons and variable exons on the ENCODE4 data.

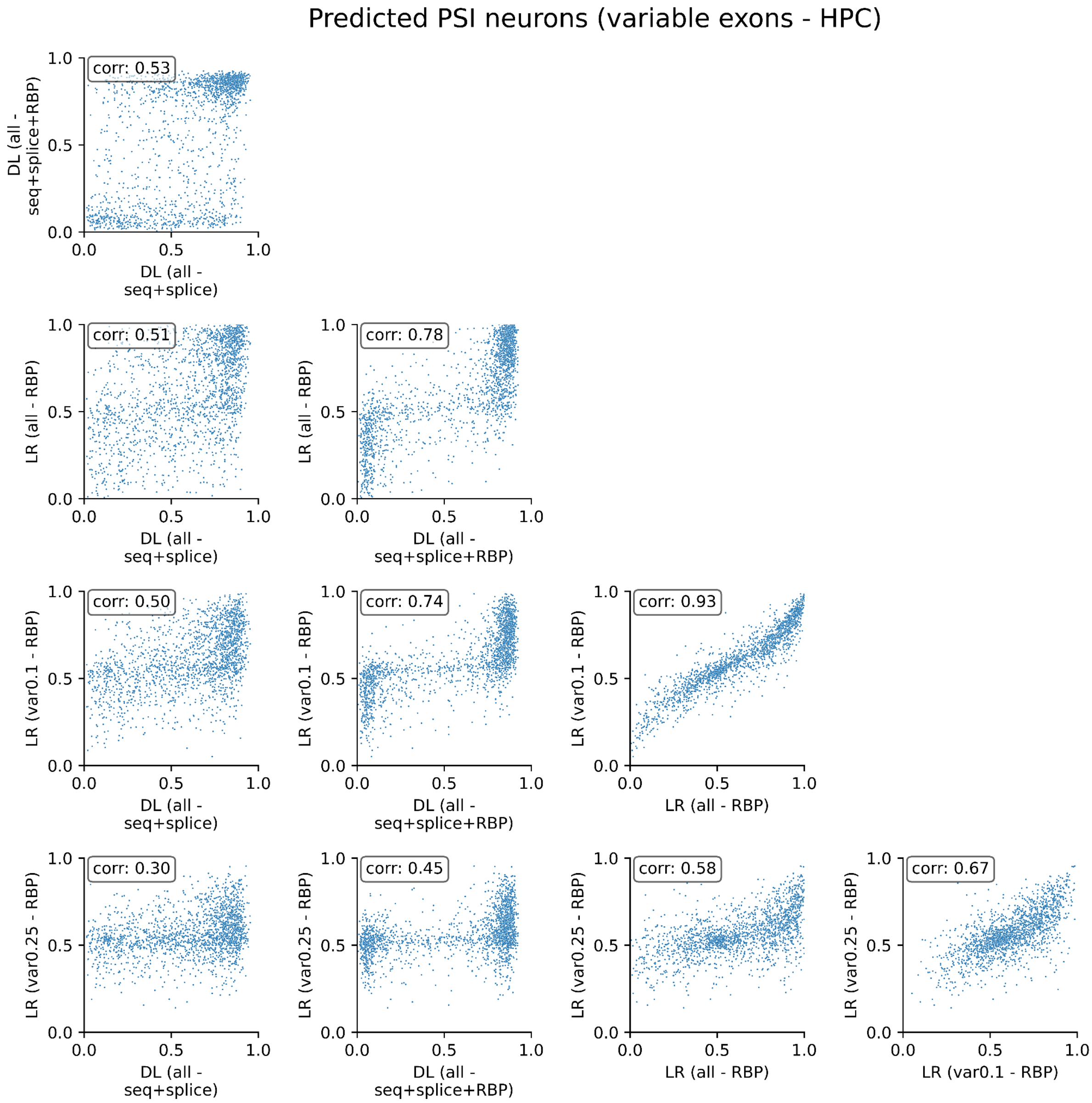

Figure S12 Scatterplots showing the agreement between models' predictions for variable exons in neurons in the hippocampus. Spearman correlation is shown in the top left corner.

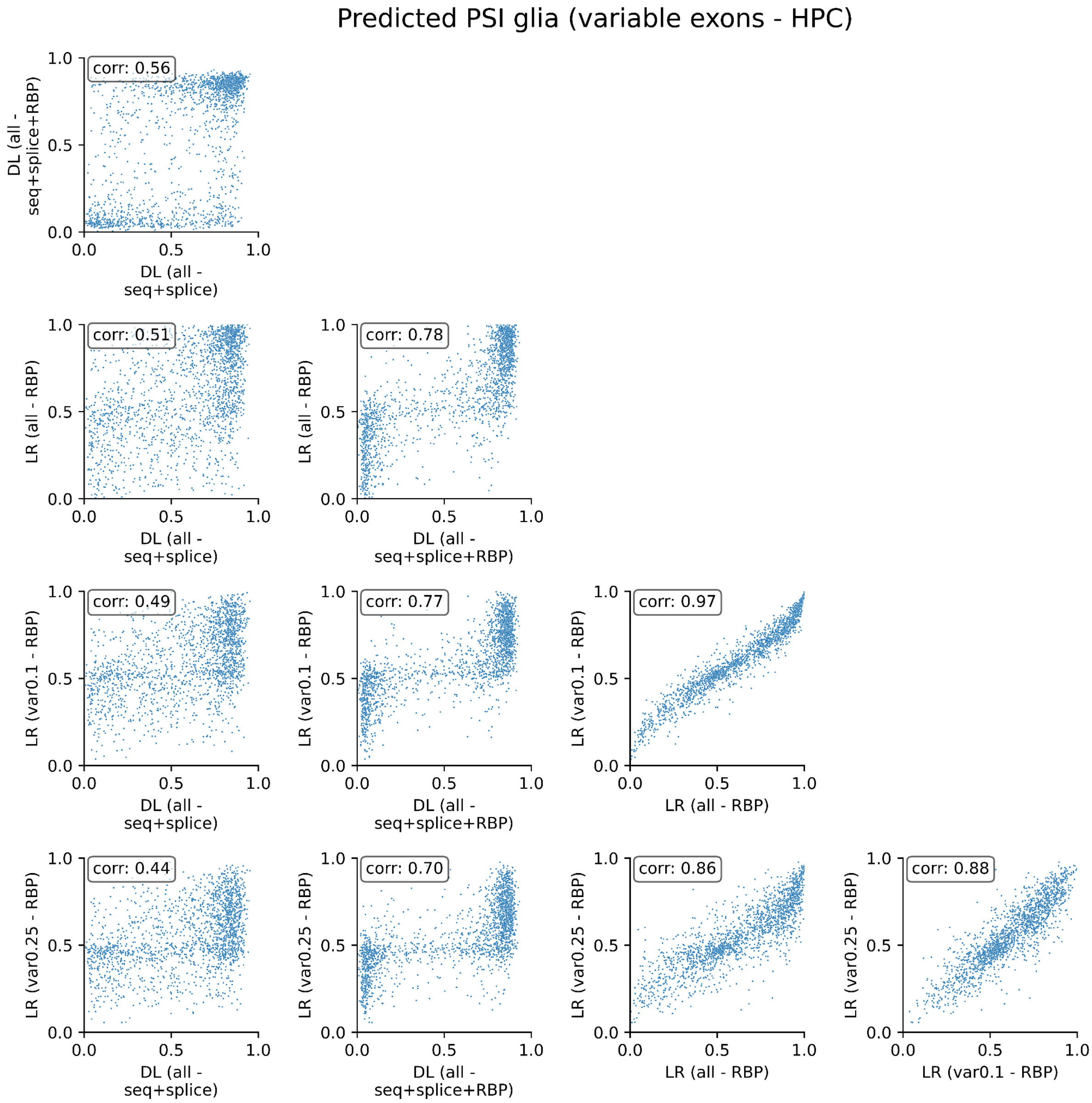

Figure S13 Scatterplots showing the agreement between models' predictions for variable exons in glia in the hippocampus. Spearman correlation is shown in the top left corner.

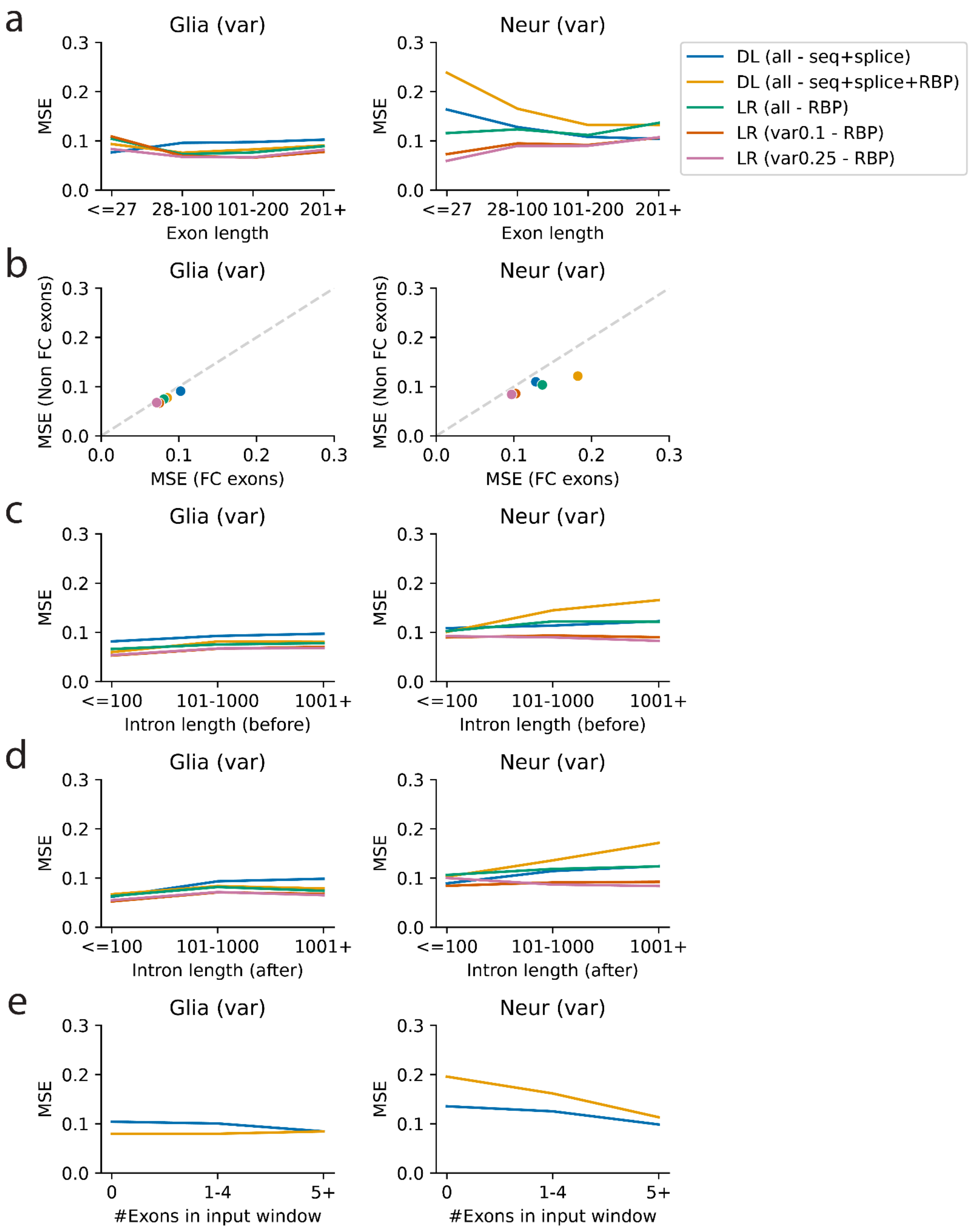

Figure S14 Performance of the models (indicated by different colors) on different subset of variable exons. Exons are split based on **a)** exon length, **b)** frame consistency (FC), **c)** intron length upstream of the exon, **d)** intron length downstream of the exon, and **e)** number of exons in the input window.

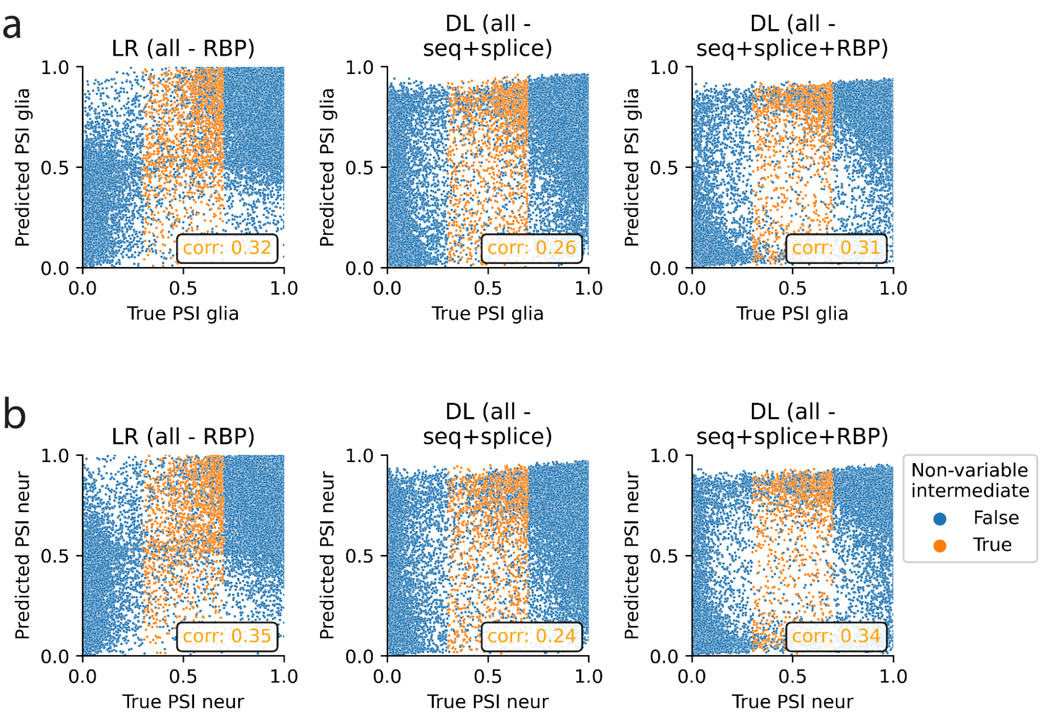

Figure S15 True versus predicted Ψ values for LR and DL models trained on all exons for **a)** glia and **b)** neurons. Non-variable intermediate exons (0.3 < Ψ < 0.7) are plotted in orange. Spearman correlation on this subset of exons is indicated at the bottom right of each plot.

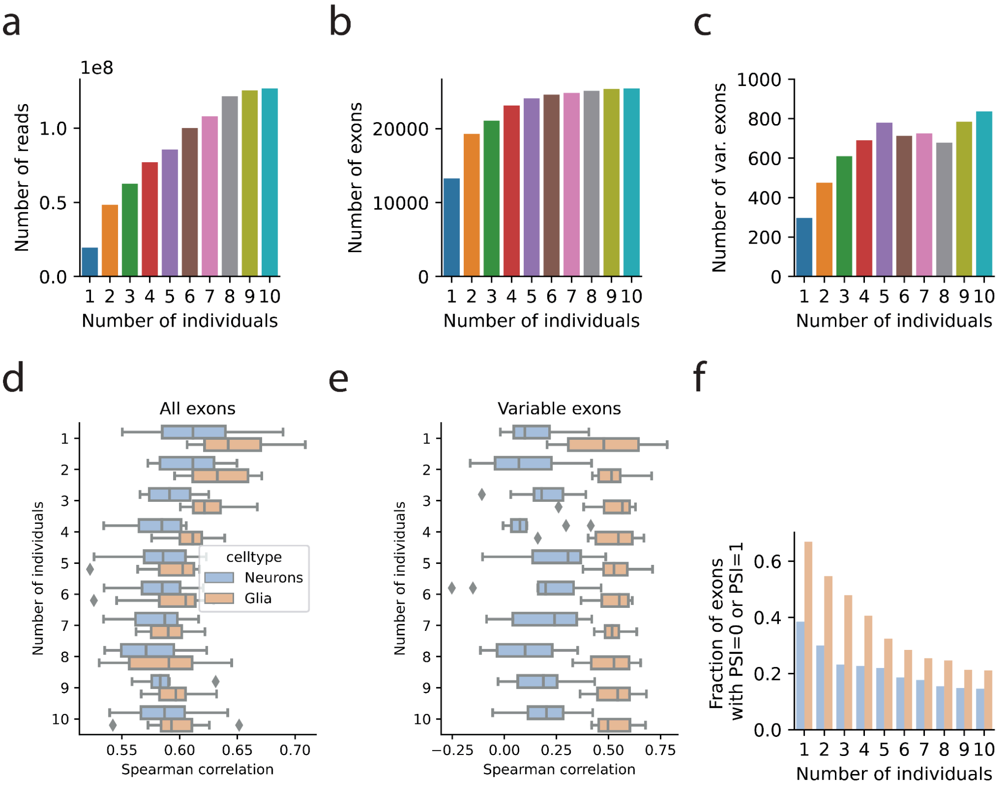

Figure S16 **a)** Number of reads, **b)** measured exons, and **c)** variable exons in the dataset when adding more individuals. **d-e)** Performance of $LR_{var0.1}$ on **d)** all exons and **e)** variable exons when adding more individuals. **f)** Fraction of exons with a binary Ψ value when adding more individuals.

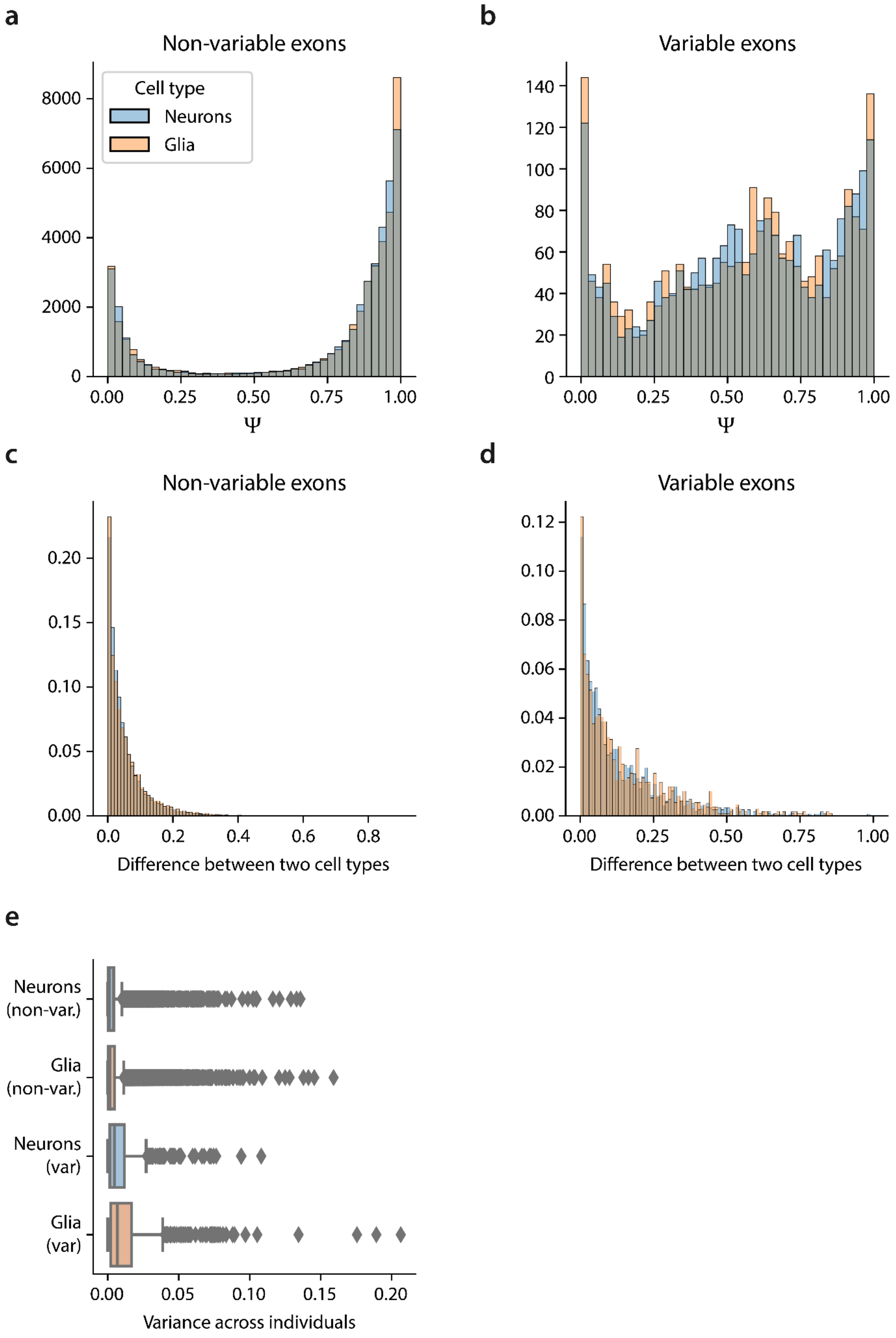

Figure S17 a-b) Distribution of Ψ split for non-variable and variable exons, **c-d)** $|\Psi_{celltype1-celltype2}|$ for the non-variable and variable exons. For neurons, inhibitory and excitatory neurons are compared. For glia, oligodendrocytes and astrocytes are compared. **e)** Variance of Ψ across the individuals.

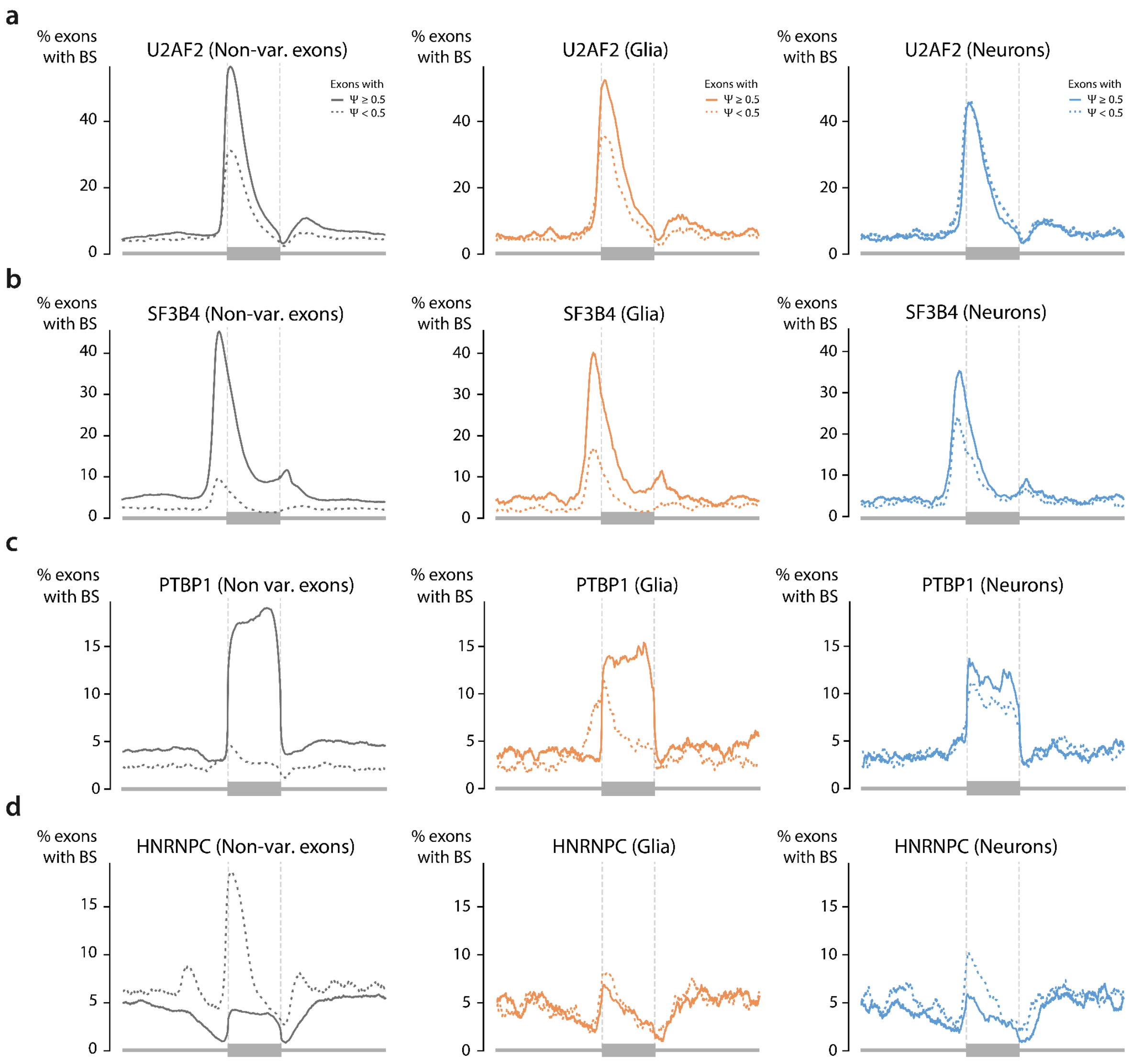

Figure S18 RBP binding profile of **a)** U2AF2, **b)** SF3B4, **c)** PTBP1, and **d)** HNRNPC in non-variable in both neurons and glia, variable exons in glia, and variable exons in neurons in the hippocampus.

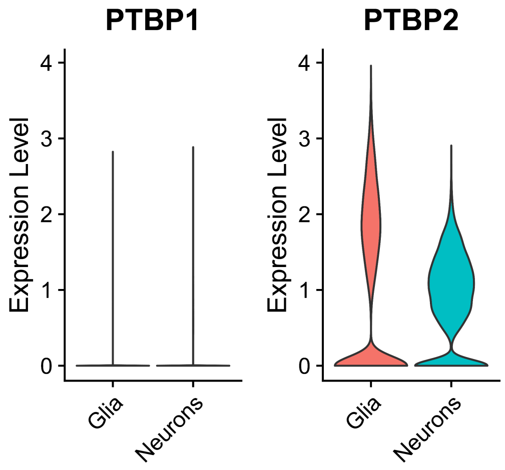

Figure S19 Expression of PTBP1 and PTBP2 in glia and neurons in the scRNA-seq hippocampus data.

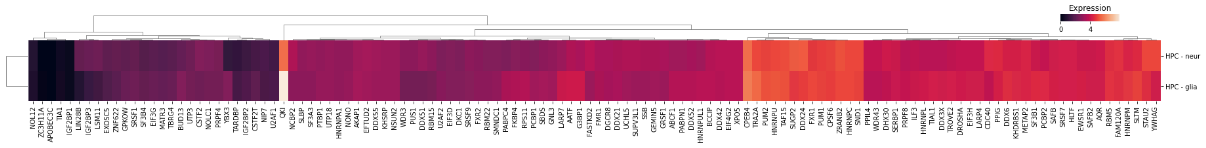

Figure S20 Expression of RBPs in the scRNA-seq data of the hippocampus.

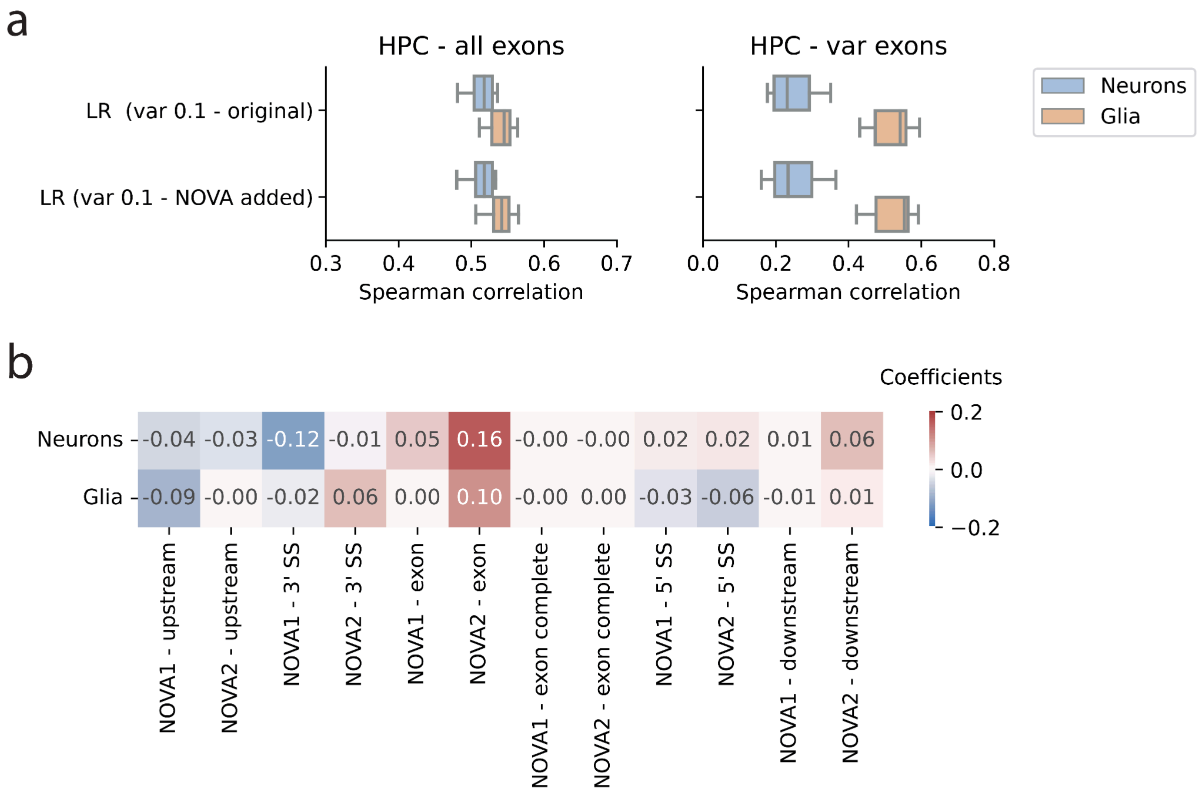

Figure S21 a) Performance of the $LR_{var0.1}$ with and without NOVA eCLIP added. **b)** Heatmap showing the coefficients for the NOVA-location features in the $LR_{var0.1}$ model trained on the ENCODE+NOVA eCLIP data.

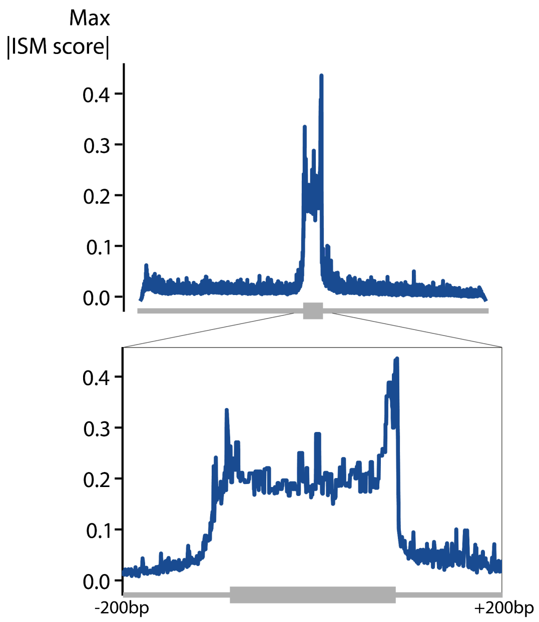

Figure S22 Maximum absolute ISM score over all sequences. Values above 0.1 are only seen in the range of 50bp upstream of the 3’ splice site until 150 downstream of the 5’ splice site. The zoomed-in plot ranges from 200bp upstream of the 3’ splice site to 200bp downstream of the 5’ splice site.

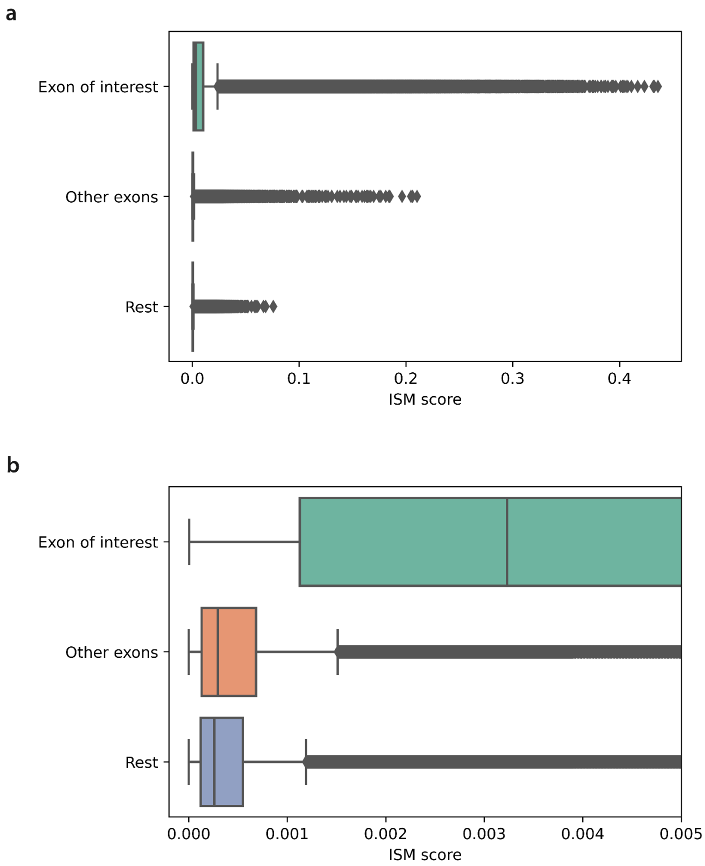

Figure S23 Boxplot showing the maximum absolute ISM score of each position. Positions are grouped based on whether they fall in the exon of interest, another exon, or the remaining sequence. **a)** Complete boxplot, **b)** zoomed in boxplot to show the difference between the other exons and the remaining sequence.

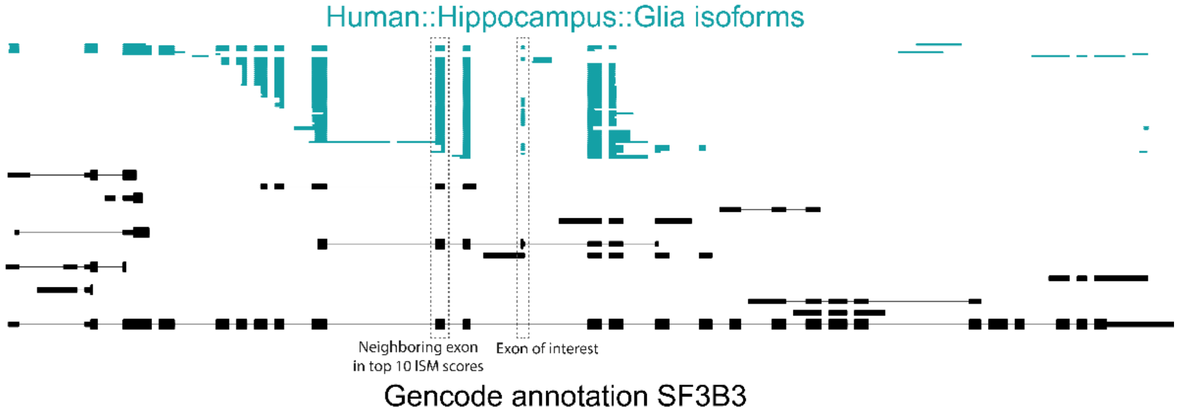

Figure S24 Potential coordination in SF3B3. If the neighboring exon is not included, the exon of interest is also not included. A mutation in the neighboring exon that decreases its Ψ value, could thus decrease the Ψ of the exon of interest as well.

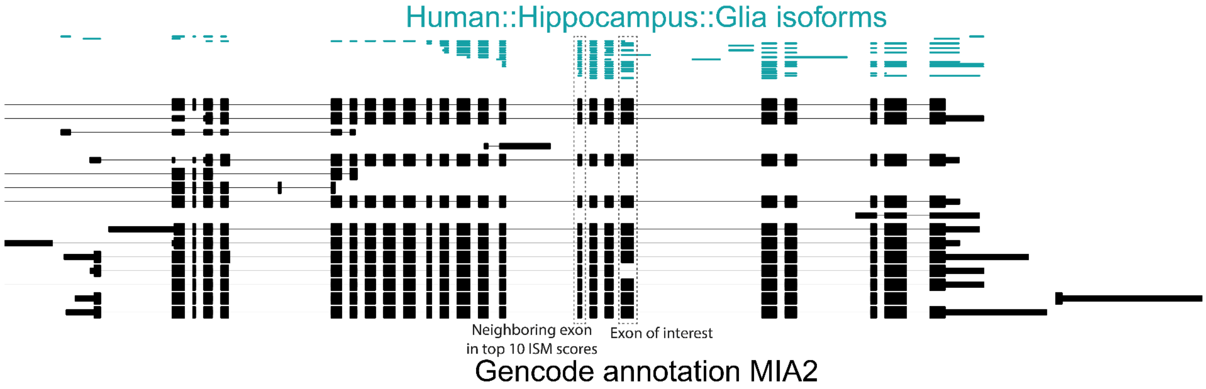

Figure S25 Potential coordination of MIA2. If the neighboring exon is included, the exon of interest is also included. A mutation in the neighboring exon that decreases its Ψ value, could thus decrease the Ψ of the exon of interest as well.

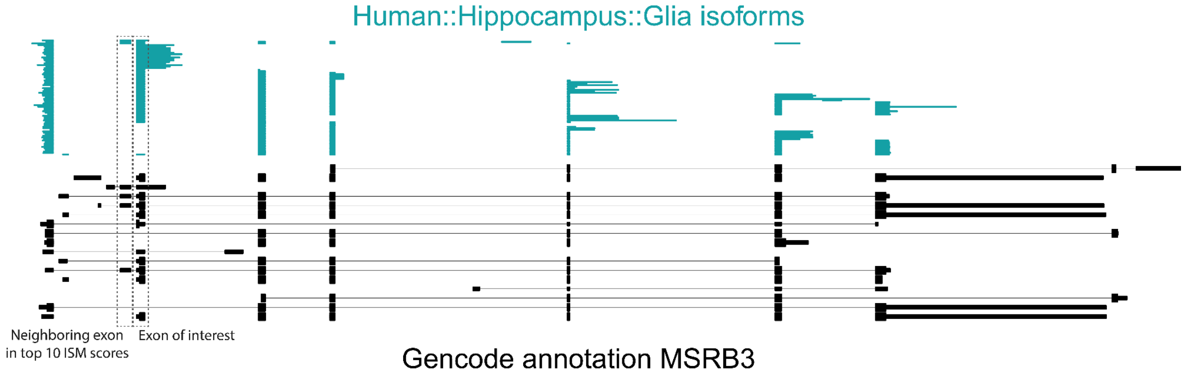

Figure S26 Potential coordination in MSRB3. The Ψ value of the neighboring exon is low, but if this exon is included, the exon of interest is also included. Especially a mutation that increases the inclusion of the neighboring exon, could potentially increase the inclusion of the exon of interest as well.

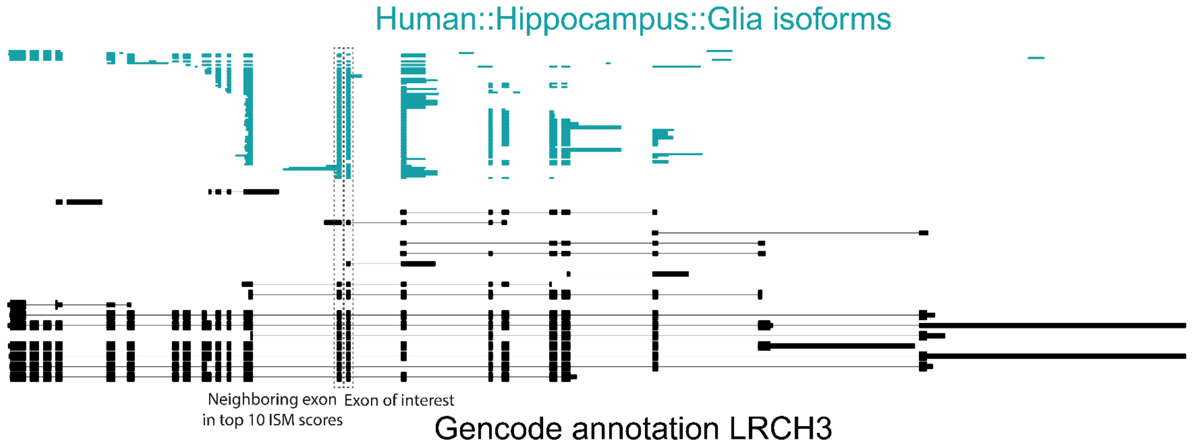

Figure S27 Potential coordination in LRCH3. The exon of interest and the neighboring exon are either included together or not.

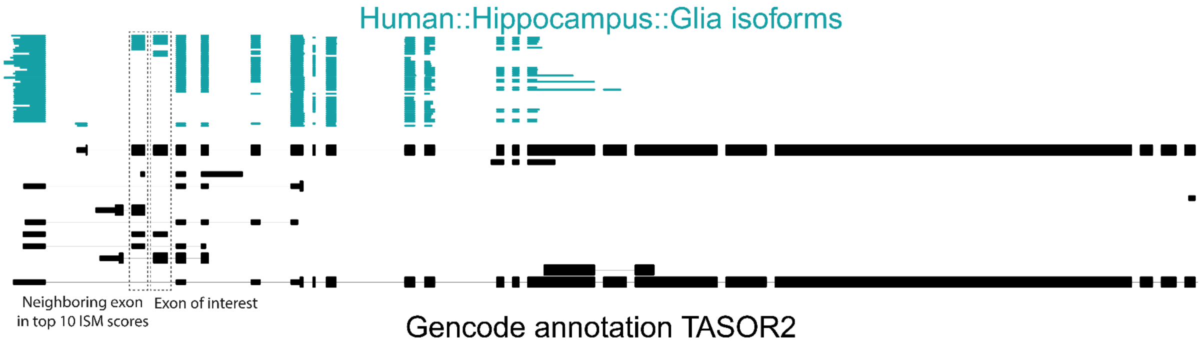

Figure S28 Here it looks random whether the neighboring exon and the exon of interest are included simultaneously, so no coordination.

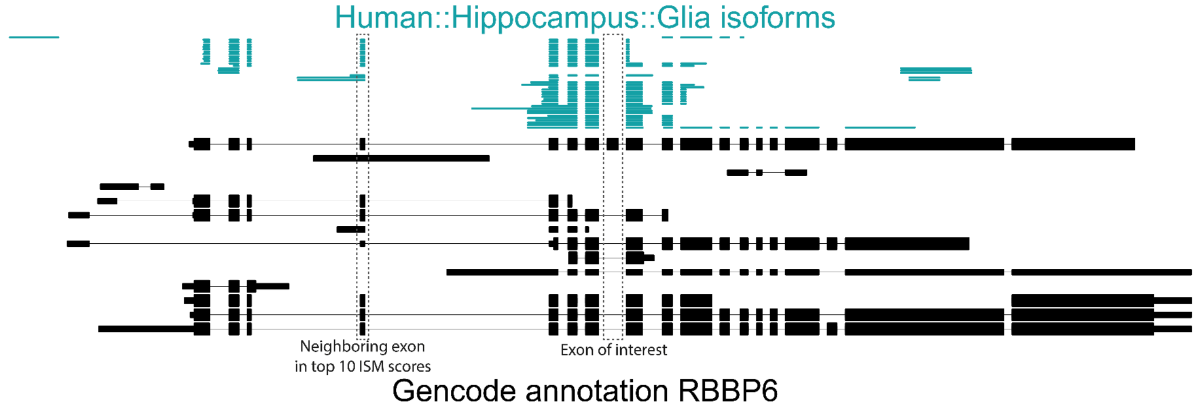

Figure S29 The exon of interest is never included in our data in glia.

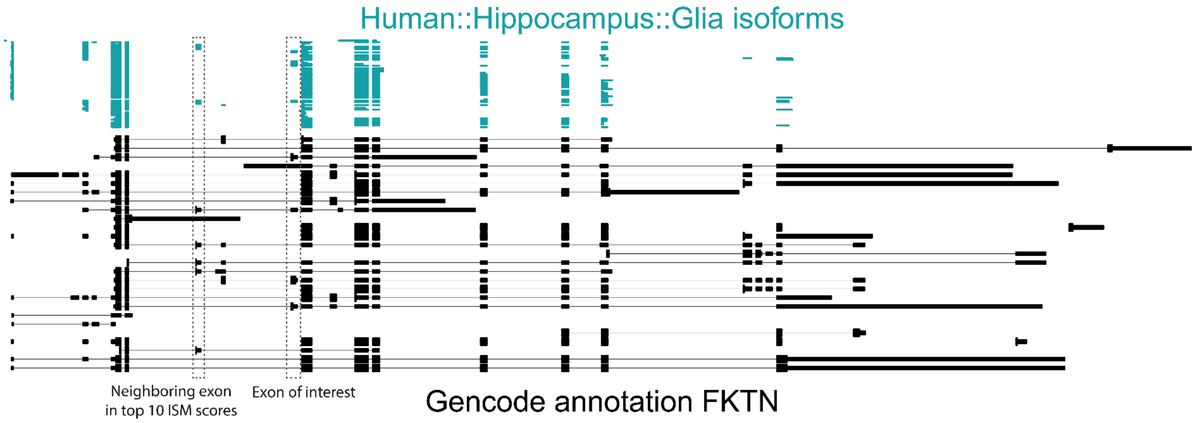

Figure S30 Here it looks random whether the neighboring exon and exon of interest are included separately or both.

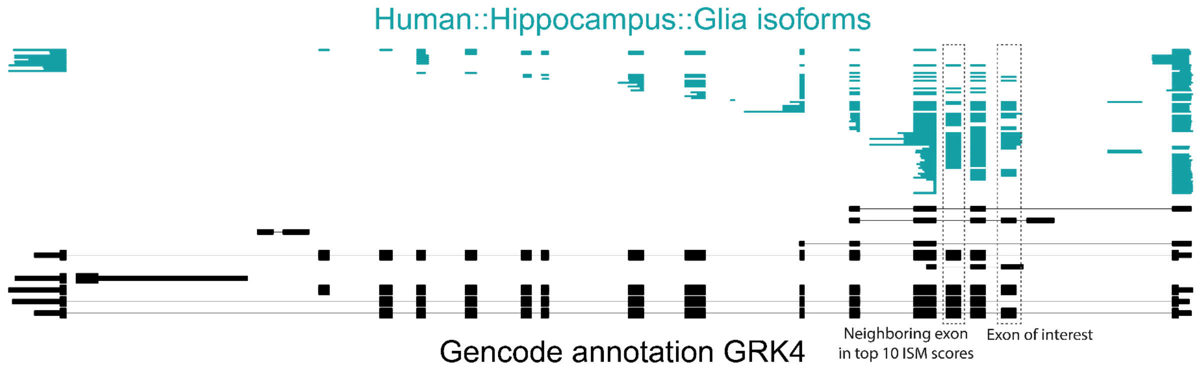

Figure S31 Here it looks random whether the neighboring exon and exon of interest are included separately or both.

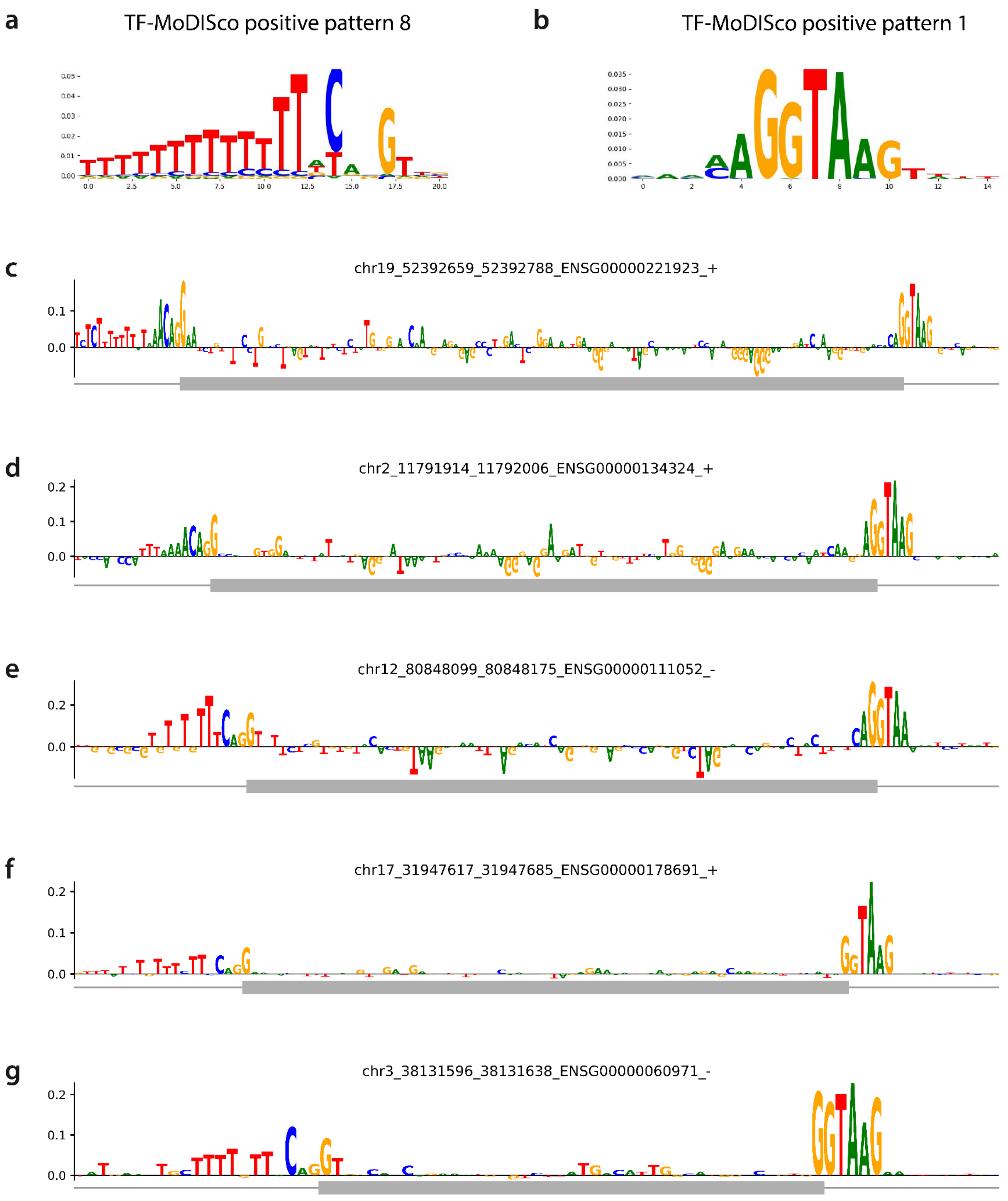

Figure S32 a-b) Example TF-MoDISco motifs that are related to the splice sites, **c-g)** ISM results of some sequences with these motifs.

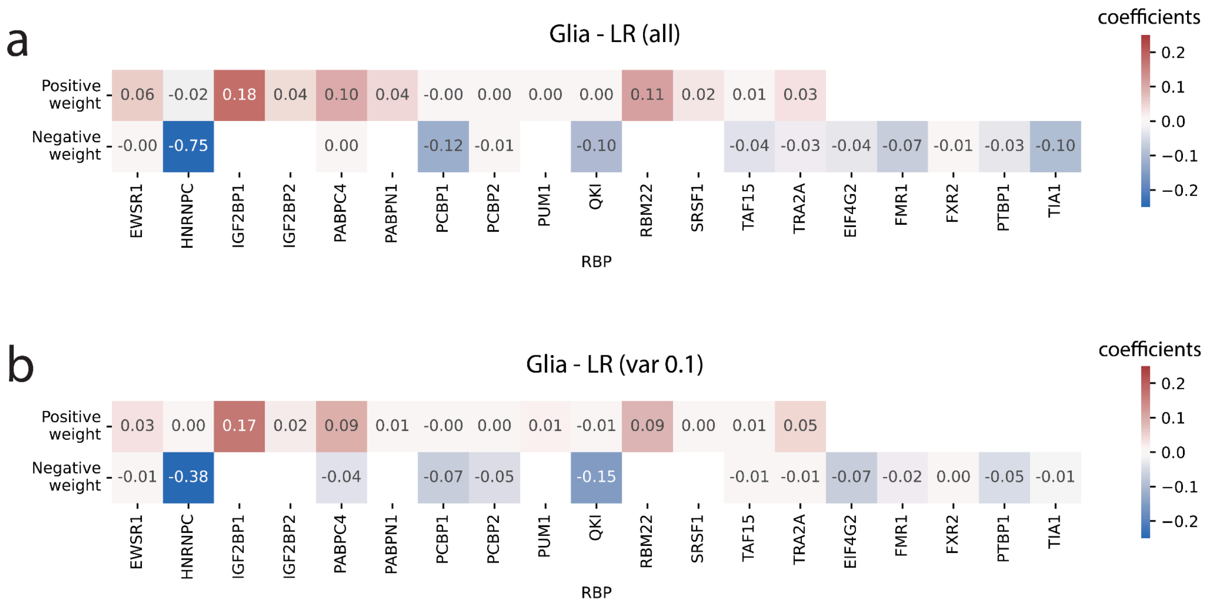

Figure S33 Consistency between the interpretation of the $DL_{all,\ seq}$ model trained on glia and **a)** LR_{all} and **b)** $LR_{var0.1}$. The rows indicate whether an RBP was assigned a positive or negative weight by TF-MoDISco. Tiles are colored by the maximum (for positive weight) or minimum (for negative weight) coefficients in the LR model.

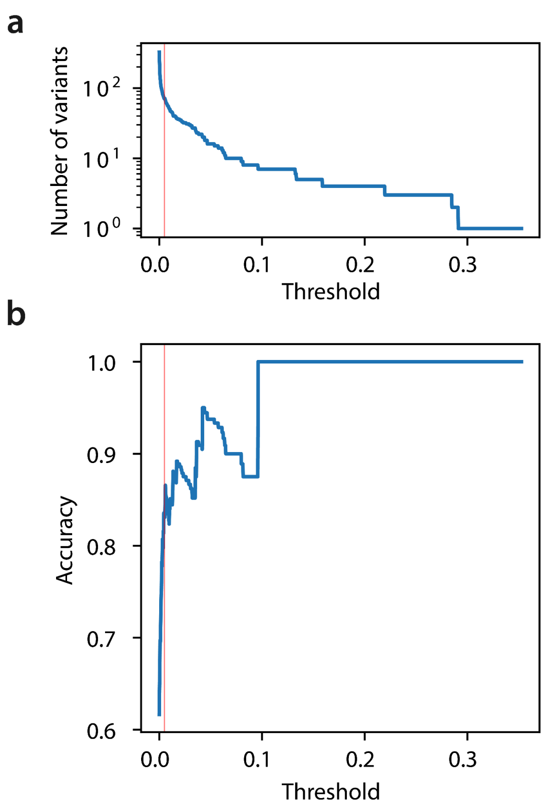

Figure S34 a) Number of variants for which the predicted effect is above the threshold, **b)** Accuracy for those variants.

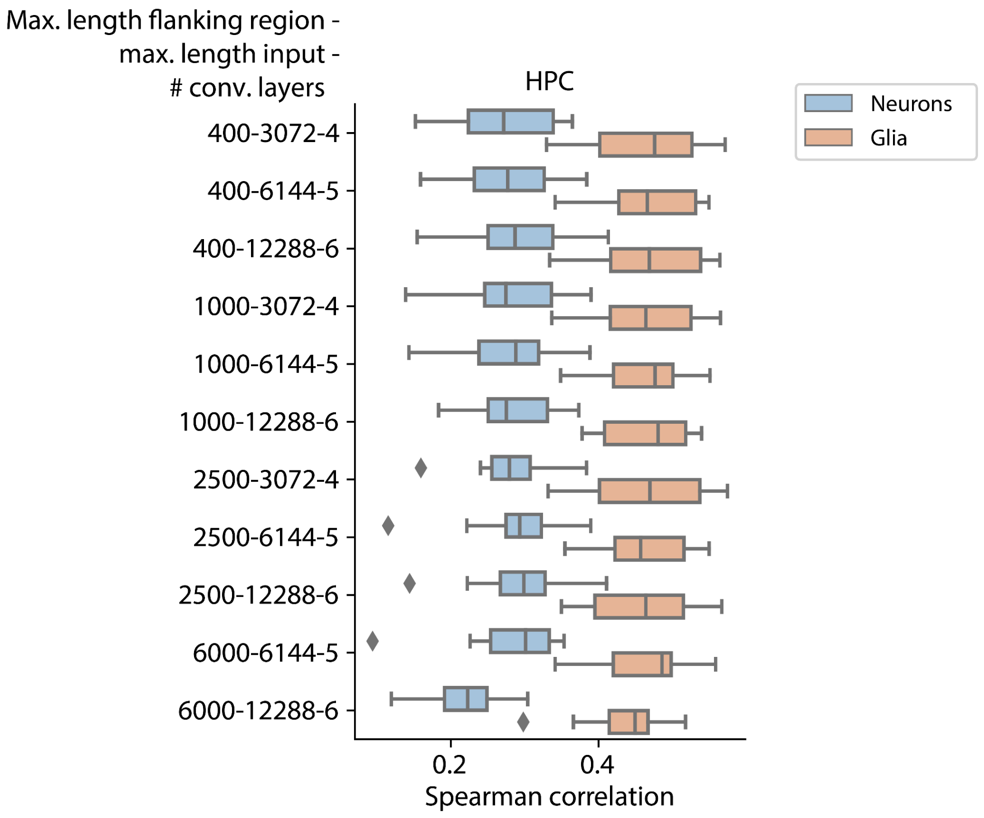

Figure S35 Performance of the DL_{all} models during the 10-fold cross-validation on variable exons from neurons and glia in the HPC. The models have different maximum lengths of flanking regions (so the sequence up- and downstream of the exon), maximum lengths of input sequences, and a different number of convolutional layers.

Table S1 Number of exons in the training data for the different models. We split the training data into ten folds during the cross-validation. Exons are always in the same folds to allow for model comparison.

	DL_{all}	LR_{all}	$DL_{var0.1}$	$LR_{var0.1}$	$LR_{var0.25}$
HPC	42,942	37,382	9,929	8,422	1,827
FC	15,747	14,077	2,637	2,315	802

Table S2 Number of measured exons (exons for which at least 10 reads were sequenced in both neurons and glia) and variable exons ($|\Delta\Psi_{glia-neur}| > 0.25$) in the hippocampus (HPC) and visual cortex (VisC) mouse data.

Brain region	Number of mice	Exons measured in both glia and neurons	Variable exons	Reference
HPC	2	23,857	528	Joglekar et al. [25]
VisC	2	48,515	1,404	Joglekar et al. [25]

Table S3 Number of measured exons (exons for which at least 10 reads were sequenced in both excitatory neurons and astrocytes) and variable exons ($|\Delta\Psi_{astro-exc.neur}| > 0.25$) in the ENCODE4 data.

Sample	Number of replicates	Exons measured in both astro and exc. neurons	Variable exons	Reference
PGP1-derived cells (in vitro differentiated)	2	28,978	205	Reese et al. [37]

Table S4 Differentially spliced RBPs

**Gene**	**Chr**	**Strand**	**Start**	**End**	**HPC - neur**	**HPC - glia**
PABPC4	1	-	39563836	39563922	0.47	0.87
			39564686	39564773	0.66	0.92
ZRANB2	1	-	71065678	71065752	0.64	0.33
PUM2	2	-	20278583	20278819	0.22	0.36
CPEB4	5	+	173943026	173943049	0.66	0.34
DROSHA	5	-	31521123	31521215	0.94	0.67
XPO5	6	-	43549489	43549578	0.71	1.00
			43549893	43549934	0.72	1.00
			43558501	43558591	0.48	1.00

TBRG4	7	-	45103333	45103443	0.94	0.64
			45104099	45104256	0.83	0.39
			45104538	45104709	0.67	0.40
			45105441	45105764	0.93	0.64
YBX3	12	-	10709908	10710114	0.92	0.57
PUS1	12	+	131939173	131939275	0.98	0.71
TAF15	17	+	35833907	35833941	0.63	0.90
EFTUD2	17	-	44854556	44854682	0.56	0.93
			44854918	44855004	0.50	0.92
			44857075	44857157	0.68	0.94
			44859905	44860045	0.63	0.90
			44863655	44863782	0.47	0.92
FXR2	17	-	7594238	7594347	0.69	1.00

Table S5 Mapping of exon names to genomic coordinates. We used reference genome GRCh38 to get the genomic coordinates and GENCODE v35 to count exons.

**Gene name**	**Chr**	**Strand**	**Exon ID**	**Start**	**End**
XRN2	20	+	Exon 21	21354789	21354872
			Exon 22	21356080	21356177
			Exon 24	21357736	21657792
TPCN1	12	+	Exon 42	113278189	113278237
ZNF880	19	+	Exon 15	52383665	52383764
RARS1	5	+	Exon 25	168497947	168498046
